# Familial episodic limb pain in kindreds with novel Nav1.9 mutations

**DOI:** 10.1371/journal.pone.0208516

**Published:** 2018-12-17

**Authors:** Risako Kabata, Hiroko Okuda, Atsuko Noguchi, Daiki Kondo, Michimasa Fujiwara, Kenichiro Hata, Yoshifumi Kato, Ken Ishikawa, Manabu Tanaka, Yuji Sekine, Nozomi Hishikawa, Tomoyuki Mizukami, Junichi Ito, Manami Akasaka, Ken Sakurai, Takeshi Yoshida, Hironori Minoura, Takashi Hayashi, Kohei Inoshita, Misayo Matsuyama, Noriko Kinjo, Yang Cao, Sumiko Inoue, Hatasu Kobayashi, Kouji H. Harada, Shohab Youssefian, Tsutomu Takahashi, Akio Koizumi

**Affiliations:** 1 Department of Health and Environmental Sciences, Graduate School of Medicine, Kyoto University, Kyoto, Japan; 2 Laboratory of Molecular Biosciences, Graduate School of Medicine, Kyoto University, Kyoto, Japan; 3 Department of Pediatrics, Akita University School of Medicine, Akita, Japan; 4 Department of Pediatrics, National Hospital Organization Fukuyama Medical Center, Hiroshima, Japan; 5 Department of Maternal-Fetal Biology, National Center for Child Health and Development, Tokyo, Japan; 6 Zen Family Clinic, Kanagawa, Japan; 7 Department of Pediatrics, Iwate Medical University School of Medicine, Iwate, Japan; 8 Division of General Pediatrics, Saitama Children’s Medical Center, Saitama, Japan; 9 Department of General Pediatrics, Shizuoka Children’s Hospital, Shizuoka, Japan; 10 Department of Neurology, Okayama University Graduate School of Medicine, Dentistry and Pharmaceutical Sciences, Okayama, Japan; 11 Department of Pediatrics, National Hospital Organization Kumamoto Medical Center, Kumamoto, Japan; 12 Center for Child Development, Hokkaido Social Welfare Corporation Taiyo no Sono, Hokkaido, Japan; 13 Department of Pediatrics, The JIKEI University School of Medicine, Tokyo, Japan; 14 Department of Pediatrics, Kyoto University Graduate School of Medicine, Kyoto, Japan; 15 Pediatric Intensive Care Unit, Nagano Children’s Hospital, Nagano, Japan; 16 Department of Developmental Medicine, Nishikawa Clinic, Yamaguchi, Japan; 17 Department of Neurology, Yanagawa Rehabilitation Hospital, Fukuoka, Japan; 18 Department of Pediatrics, Miyazaki University Hospital, Miyazaki, Japan; 19 Department of Pediatrics, Graduate School of Medicine, University of the Ryukyus, Okinawa, Japan; 20 Department of Preventive Medicine, St. Marianna University School of Medicine, Kanagawa, Japan; 21 Department of Biomedical Sciences, College of Life and Health Science, Chubu University, Aichi, Japan; Boston Children’s Hospital and Harvard Medical School, UNITED STATES

## Abstract

We previously performed genetic analysis in six unrelated families with infantile limb pain episodes, characterized by cold-induced deterioration and mitigation in adolescence, and reported two new mutations p.R222H/S in *SCN11A* responsible for these episodes. As no term described this syndrome (familial episodic pain: FEP) in Japanese, we named it as”小児四肢疼痛発作症”. In the current study, we recruited an additional 42 new unrelated Japanese FEP families, between March 2016 and March 2018, and identified a total of 11 mutations in *SCN11A*: p.R222H in seven families, and p.R225C, p.F814C, p.F1146S, or p.V1184A, in independent families. A founder mutation, *SCN11A* p.R222H was confirmed to be frequently observed in patients with FEP in the Tohoku region of Japan. We also identified two novel missense variants of *SCN11A*, p.F814C and p.F1146S. To evaluate the effects of these latter two mutations, we generated knock-in mouse models harboring p.F802C (F802C) and p.F1125S (F1125S), orthologues of the human p.F814C and p.F1146S, respectively. We then performed electrophysiological investigations using dorsal root ganglion neurons dissected from the 6–8 week-old mice. Dissected neurons of F802C and F1125S mice showed increased resting membrane potentials and firing frequency of the action potentials (APs) by high input–current stimulus compared with WT mice. Furthermore, the firing probability of evoked APs increased in low stimulus input in F1125S mice, whereas several AP parameters and current threshold did not differ significantly between either of the mutations and WT mice. These results suggest a higher level of excitability in the F802C or F1125S mice than in WT, and indicate that these novel mutations are gain of function mutations. It can be expected that a considerable number of potential patients with FEP may be the result of gain of function *SCN11A* mutations.

## Introduction

Familial episodic limb pain is clinically characterized by paroxysmal pain episodes that appears during infancy, gradually decreases with age, and are often induced by fatigue, bad weather or cold temperature [1–3]. As there was no appropriate name describing this syndrome in Japanese, we designated this as小児四肢疼痛発作症, which corresponds to familial episodic pain (FEP) [1].

In our previous study, we identified *SCN11A* p.R222H and p.R222S in six unrelated Japanese families with FEP, and determined them as founder mutations in the Tohoku area of northern part of mainland Japan. We also demonstrated the pathological role of p.R222S in FEP, using a knock-in mouse model combined with behavioral and electrophysiological investigations [3].

*SCN11A* encodes Nav1.9, a TTX resistant subtype of voltage gated sodium channels (Nav), which contributes to the generation of a persistent inward current at subthreshold voltages [4]. Nav1.9 together with the Nav1.7, Nav1.8 subtypes are strongly expressed in sensory neurons and have been associated with various human pain disorders [5–13]. Indeed Nav1.9 is associated with diverse clinical disorders including familial episodic limb pain [1–3, 14], congenital insensitivity to pain [15–18], and small fiber neuropathy [19–21]. It is particularly interesting that Nav1.9 channelopathy is often reported to be accompanied by autonomic symptoms such as hyperhidrosis and/or gastrointestinal dysfunction [1–3, 14–20].

In our previous study [3], we investigated patients with FEP in limited local areas, mainly in the northern part of Japan. It therefore remained unknown whether FEP is broadly distributed throughout Japan, and whether additional Nav1.9 variants exist among the other FEP patients. In the present study, we further extended our research area to nationwide, and found that FEP patients carrying the Nav1.9 p.R222H mutation are more frequently distributed in the Tohoku area than in other parts of Japan. Additionally, we identified two novel Nav1.9 variants (p.F814C, p.F1146S) in two multigenerational pedigrees of the 42 independent pedigrees of FEP Japanese families that we investigated. To determine the functions of these novel variants, we generated knock-in mice harboring these mutations, and found that these missense mutations are associated with hyperexcitability of dorsal root ganglion (DRG) neurons.

## Results

### Genetic analysis of *SCN11A* in families with FEP

**Fig 1:**
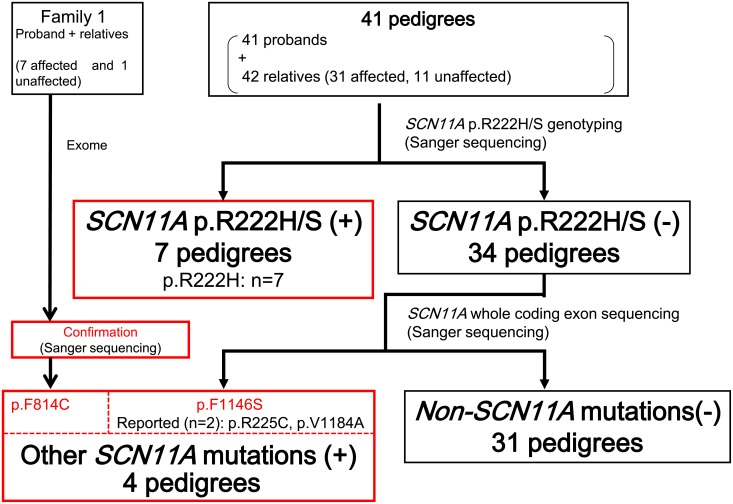
Scheme of *SCN11A* mutation screening.

By screening for the p.R222H or p.R222S variants, 7 pedigrees out of the 42 pedigrees analyzed were found to carry the p.R222H mutation (16.7%, 7/42pedigrees) (Fig 1) (Table 1).

**Table 1:** Summary of genetic tests for FEP.

Residential Area	Number of Pedigrees	FEP without Nav1.9 mutation	Numbers of FEP with Nav1.9 mutation		Mutation
			with p.R222H	with other mutation	
Hokkaido	1	1	0	0	
Tohoku	15	10	5	0	
Kanto	11	9	1	1	p.F1146S
Chubu	2	1	1	0	
Kansai	6	6	0	0	
Chugoku/Shikoku	3	1	0	1	p.F814C
				1	p.R225C [1]
Kyushu	3	2	0	1	p.V1184A [2]
Okinawa	1	1	0	0	
Total (%)	42	31 (73.8)	7 (16.7)	4 (9.5)	

**Fig 2:**
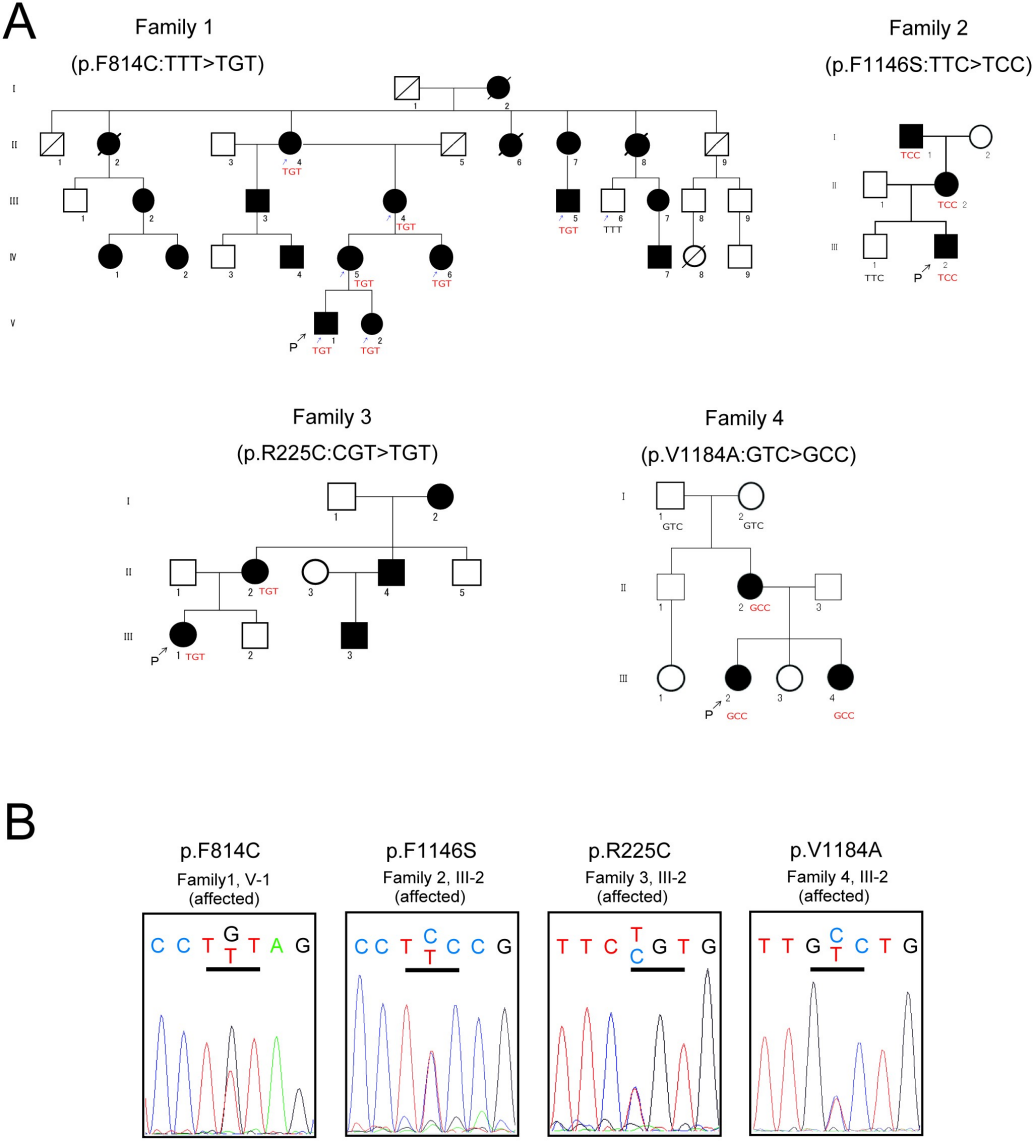
Pedigrees of Japanese familial episodic pain syndrome families. (A) Black and white symbols indicate affected and unaffected individuals, respectively. Squares and circles indicate males and females, respectively. Diagonals indicate deceased individuals. “P” indicates the proband. In family 1, small arrows indicate individuals selected for exome sequencing. For each family, codons revealed by sequencing are shown under the individual symbols of those who participated in the genetic analysis. (B) Sequence chromatographs of the identified *SCN11A* mutations.

By exome analysis and subsequent confirmation by Sanger sequencing, we found a novel *SCN11A* mutation (p.F814C) in Family 1, which was in complete concordance with the affection status: among the individuals who participated in the mutation analysis, 7 affected members carried this mutation, whereas the unaffected member did not (Fig 2). Within the remaining 34 pedigrees, we also identified one novel mutation (p.F1146S), as well as two previously-known mutations (p.R225C [1], p.V1184A [2]) (Table 1) (Figs 1 and 2).

The 1000 genomes database showed that neither of these two novel mutations (p.F814C and p.F1146S) were found in the Japanese population or in other populations. These two mutations were also not included in another Japanese variant database, the Human Genetic Variation Database. Sequence alignment of Nav family members indicated that the phenylalanine at position 814 of *SCN11A* was highly conserved with other Nav members, whereas phenylalanine at 1146 was not conserved (S1 Fig).

Therefore, of the 42 pedigrees analyzed, a total of 11 pedigrees carried an *SCN11A* mutation (26.2%, 11/42 pedigrees). However, among the 31 pedigrees in which we could not detect any *SCN11A* mutations, there were some differences in the clinical symptoms: two pedigrees (one from Kanto and one from Okinawa) had a later onset of pain episodes (onset after the age of 8) than the other pedigrees (onset before the age of 6), while two pedigrees from the Kansai area had pain episodes that were not only localized to the limbs but also to the body trunk. When these four pedigrees are excluded from the analysis, the *SCN11A* mutation-positive pedigrees amounts to 11 of 38 pedigrees (28.9%, 11/38 pedigrees).

Table 1 shows the geographical distribution of the 42 pedigrees throughout Japan. The p.R222H mutation was most frequently observed in the Tohoku area (5 out of 7 pedigrees), while one pedigree was found in the Chubu area and another pedigree in the Kanto area. The great-grandfather of the proband of the Chubu p.R222H-positive family, who also had FEP, was from the Tohoku area. The mother of the proband of the Kanto p.R222H-positive family, whose parents were from the Tohoku area, also had limb pain episodes during her childhood. Other additional mutations were identified in various areas (p.F1146S in the Kanto area; p.F814C and p.R225C in the Chugoku/Shikoku area; and p.V1184A in the Kyushu area), except in the Tohoku area.

### Phenotypic characterization

The clinical features of the pedigrees carrying the Nav1.9 mutations are described below (Table 2).

**Table 2:** Clinical manifestations of FEP.

References+A1:H4A1:H9	Zhang et al. [1] (2013)	Leipold et al. [2] (2015)	Okuda et al. [3] (2016)	This study			
**Mutation types**	p. R225C and p.A808G	p.V1184A	p.R222H and p.R222S	p.F814C	p.F1146S	p.R225C	p.V1184A
**Number of pedigrees (or patients)**	2 families	1 family	6 families	1 family	1 family	1 family	1 family
**Onset of pain**	1 year old	Within 1 or 1.5 years	Infancy	Within 2 to 3 years	Infancy	Infancy	1 year old
**Location of pain**	Principally to the lower extremities. In a few adult patients, pain located at palms, wrists, soles and knees	Lower extremities, occasionally upper. Start in joints and radiates to arms and legs	Knees, ankles, wrists and elbows in frequency order. Occasionally localized to forearms, brachia, palms, fingers, thighs, and acrotarsia	Lower and upper extremities. Often located to forearms, brachium, thigh and sural region	Elbows, toes, and knees. Occasionally localized to forearms	Upper and lower extremities. Often located to forearms, thighs and foot arches	Lower and upper extremities. Often located to fingers, toes, and knees
**Triggers of pain**	Rainy days, weakness, fatigue, illness	Gluten, cold temperature, exhaustion, illness	Weather change, cold temperature, fatigue	Fatigue	Rainy days, cold temperature, fatigue	Cold temperature, fatigue	Cold temperature, fatigue
**Frequency of pain**	6–8 times/month	2-3times/month	About 10 times/month (unstable)	10–20 times/month	About 10 times/month	About 10 times/month (unstable)	About 15 times/month
**Duration of pain**	15–20 min	20–30 min	15–30 min (~2 hours)	30 min	30 min	20 min (~2.5 hours)	20 min
**Response to medication**	Ibuprofen	Ibuprofen, Naproxen, Colchicine	Acetaminophen, Ibuprofen, Loxoprofen	Acetaminophen, Ibuprofen, Loxoprofen	None	Loxoprofen, Acetaminophen	Acetaminophen
**Painful time of the day**	Late in the day	Late afternoon, early evening or night	Occasionally during night, while asleep	Occur more frequently during night time and early morning than daytime	Occur more frequently during night than daytime	Not related to time	Occur more often in late afternoon, early evening, or night
**Cold feeling**	Yes	NR	Yes	NR	NR	Yes	No
**Burning sensation**	NR	Yes	No	Yes	NR	No	No
**Autonomic symptoms**	Sweating	Constipation (since age of 18), diarrhea	Anorexia and diarrhea following the end of a series of pain episodes in a few patients	Constipation (occurred since around age of 3 but disappeared after a few years)	Lower abdominal pain or flatulence during limb pain	None	Constipation (since around age of 16)
**Skin changes**	NR	Flushing of the neck and face	No	No	No	NR	No
**Measures for pain relief**	Hot compress, pressure	Warmth, gluten-free diet	Warmth, massage, compress	Warmth, pressure	Warmth, pressure	Warmth, pressure	Warmth, massage
**Age-related changes of pain**	Decreased with age	Decreased with age	Decreased with age (since around age of 15)	Decreased with age (since around age of 40)	Decreased with age (since around the age of 15)	Decreased with age (since around age of 20)	Decreased with age (since around age of 14)
**Development**	Normal	Normal	Normal	Normal	Normal	Normal	Normal

Clinical characteristics correspond with Nav1.9 mutations; NR, indicates was not described in these reports; Yes/No indicates the presence of symptoms.

#### Family 1 (p.F814C)

The proband (V-1) is a 6-year-old boy, who complained of typical infantile pain episode symptoms from around 2 years of age. The pain occurs in the lower or upper extremities, often in the forearms, brachia, thighs or sural regions. Pain episodes are primarily induced by fatigue, and are not aggravated by bad weather or cold temperature. His younger sister (V-2, 5-years-old), mother (IV-5, 30-years-old), and grandmother (III-4, 55-years-old) also have episodic pain symptoms. The grandmother has much less frequent pain episodes after she reached her 40s, but they have not completely disappeared. The proband has suffered from constipation from the age of 3 years, but it ameliorated from around the age of 6 years, whereas other members did not have constipation. The mother and grandmother also suffer from migraine. The grandmother’s family is originally from the Chugoku area.

#### Family 2 (p.F1146S)

The proband (III-2) is a 5-year-old boy who appears to have had the typical limb pain symptoms since six months of age. The pain is localized to the elbows, toes, knees, and occasionally to forearms. The symptom is induced by rainy days, cold temperature, and also fatigue. Pain episodes occur more frequently at night than in the daytime, depriving him of sleep. His grandfather (I-1, 63-years-old) had the same symptoms until he reached 17 years of age, while his mother (II-2, 37-years-old) still has limb pain episodes, although with much less frequency than in childhood after the age of 13. In this family, patients also complain of gastrointestinal symptoms that occur simultaneously with limb pain; the mother has lower abdominal pain that occurs concomitantly with peristaltic movement, and the proband has flatulence during pain episodes. The grandfather’s family is originally from the Kanto area.

#### Family 3 (p.R225C)

This mutation was originally reported in a Chinese pedigree [1]. The proband (III-1) is a 17-year-old female, who appears to have had limb pain episodes since infancy. Her mother (II-2, 44-years-old), mother’s brother (II-4, 42-years-old), the son of the mother’s brother (III-3, 15-years-old), and grandmother (I-2, 70-years-old) are all also affected. The characteristics of their symptoms are mostly similar to that previously reported for a Chinese family [1], except that this Japanese family did not have hyperhidrosis. Although a previous study suggested the contribution of *SCN11A* p.R225C to the development of essential tremor [22], none of the members of this family had essential tremor. The grandmother was raised in Japan as an orphan but had heard that one of her biological parents was working as an interpreter during wartime. Although there is the possibility that she is of Chinese origin, we are unable to independently verify this at present.

#### Family 4 (p.V1184A)

This mutation was previously reported in a mixed European ancestry [2]. The proband (III-2) is a 4-year-old girl, who appears to have had limb pain episodes since she was one year old. Her younger sister (III-4, 8-months-old) and mother (II-2, 37-years-old) also have limb pain episodes. The characteristics of their symptoms are mostly consistent with that previously reported [2], although their symptoms are not affected by gluten-containing foods, nor do they have flushing of the neck and face. Similarly to the previous report [2], the 37-year-old mother has had fewer pain episodes after the age of 14, but had constipation after the age of around 16. The mother states that constipation exacerbated her limb pain. Neither of the parents of the mother carry the p.V1184A mutation, suggesting that this was the result of a de novo mutational event in the mother.

The other seven families were found to carry the p.R222H mutation, and displayed the same symptoms as reported previously [3] (Table 2).

### Characterization of DRG neurons in knock-in mice harboring the novel mutations, p.F802C or p.F1125S

Previously, we demonstrated the association of the painful phenotype with up-regulated excitability of small DRG neurons by electrophysiological analyses in Nav1.9 p.R222S mutation knock-in mice [3].

**Fig 3:**
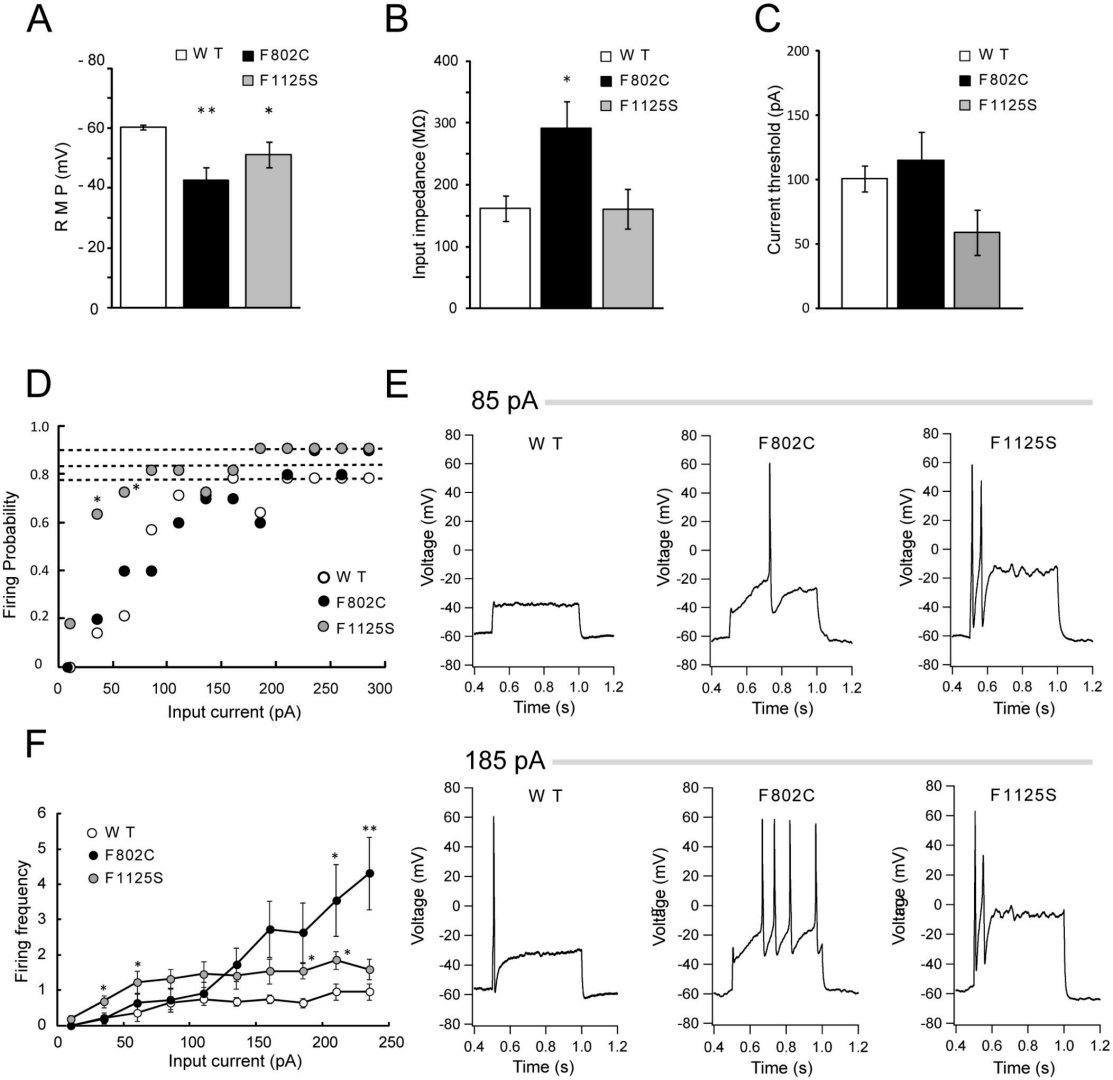
*SCN11A* mutations (F802C and F1125S) increase excitability in DRG neurons. Knock-in mice harboring the Nav1.9 mutations (F802C or F1125S) significantly depolarized the RMP compared with WT mice (WT, n = 11; F802C, n = 6; F1125S, n = 6). (B) Input impedance was measured at an injection current of 10 pA. F802C mice showed a significant increase in input impedance compared with WT mice (WT, n = 13; F802C, n = 6; F1125S, n = 9). (C) Current threshold was not significant difference among the WT, F802C, and F1125S mice (WT, n = 13; F802C, n = 10; F1125S, n = 11). (D) Comparison of firing probability between WT and each mutation. The maximum firing rate of each cells during current steps of 10–285 pA are represented by the dashed lines (WT, n = 14; F802C, n = 10; F1125S, n = 11). (E) Representative traces of the AP firing, recorded from small DRG neurons (< 25 μm) in each mutation (F802C and F1125S), show increases during 500 ms current steps of 85 pA and 185 pA. Upper and lower panels represent the response to input current 85 pA and 185 pA respectively. (F) Comparison of the repetitive number of APs between WT and each mutation (WT, n = 14; F802C, n = 11; F1125S, n = 11). The range of 500-ms-step current injections was 10–235 pA. Statistical tests were performed using one-way ANOVA followed by post-hoc Student’s *t*-test with Bonferroni correction (A, B, and C), Fisher’s exact with Bonferroni correction (D) or Kruskal-Wallis test followed by Dunn’s multiple comparisons test (F). P values were corrected by the Bonferroni method or Dunn’s multiple comparisons test. *p < 0.05 vs WT, **p < 0.01 vs WT.

In this study, we also generated knock-in mouse models harboring one of the two novel mutations (p.F802C and p.F1125S; orthologues of human p.F814C and p.F1146S, respectively). We then performed current-clamp recordings in small DRG neurons (< 25 μm) isolated from wild type (WT) mice and from F802C and F1125S knock-in mice to assess the effects of these Nav1.9 mutations on DRG neuronal excitability (Fig 3).

The RMP was significantly different when the small DRG neurons of F802C and F1125S mice were compared with WT mice (WT, -60.36 ± 0.73 mV, n = 11; F802C, -42.76 ± 4.26 mV, n = 6, p < 0.01; F1125S, -51.22 ± 4.15 mV, n = 6, p < 0.05) (Fig 3A). The input impedance of DRG neurons from F802C mice, but not from F1125S mice, was significantly higher than in WT mice when measured in response to a current injection of 10 pA (WT, 162.18 ± 14.33 MΩ, n = 13; F802C, 290.81 ± 45.29 MΩ, n = 6, p < 0.05; F1125S, 176.31 ± 30.67 MΩ, n = 9, p = 1) (Fig 3B). The current threshold was not significant difference (p>0.05 by one-way ANOVA) among the WT, F802C, and F1125S mice (WT, 100.38 ± 10.02 pA, n = 13; F802C, 115.00 ± 22.30 pA, n = 10; F1125S, 62.27 ± 17.66 pA, n = 11) (Fig 3C).We also measured several parameters of the action potential (AP) which was generated by a current injection of 185 pA (Table 3).

**Table 3:** Parameters of action potential in DRG of WT and mutants mice.

genotype	maximum rate of rise (mv/ms)	maximum rate of fall (mv/ms)	AP amplitude (mV)	AP width (ms)
WT	41.17 ± 12.07 (n = 10)	-25.00 ± 3.62 (n = 10)	102.14 ± 4.56 (n = 10)	7.19 ± 1.75 (n = 10)
F802C	44.22 ± 9.22 (n = 5)	-20.16 ± 2.89 (n = 5)	84.78 ± 10.24 (n = 5)	6.84 ± 1.02 (n = 5)
F1125S	29.82 ± 7.06 (n = 6)	-22.53 ± 5.13 (n = 6)	117.12 ± 6.59 (n = 6)	8.19 ± 1.47 (n = 6)

There were no significant differences (p>0.05 by one-way ANOVA) among the WT, F802C, and F1125S mice in the AP parameters, such as the maximum rate of rise of AP (WT, 41.17 ± 12.07 mV/ms, n = 10; F802C, 44.22 ± 9.22 mV/ms, n = 5; F1125S, 29.82 ± 7.06 mV/ms, n = 6); the maximum rate of fall of AP (WT, -25.00 ± 3.62 mV/ms, n = 10; F802C, -20.16 ± 2.89 mV/ms, n = 5; F1125S, -22.53 ± 5.13 mV/ms, n = 6); AP width (WT, 7.19 ± 1.75 ms, n = 10; F802C, 6.84 ± 1.02 ms, n = 5; F1125S, 8.19 ± 1.47 ms, n = 6) or the AP amplitudes (WT, 102.14 ± 4.56 mV, n = 10; F802C, 84.78 ± 10.24 mV, n = 5,; F1125S, 117.12 ± 6.59 mV, n = 6) (Table 3). We determined the firing probability of small DRG neurons isolated from three mouse groups by injecting constant current which was increased from 10 to 285 pA by 25 pA increments. DRG neurons isolated from F1125S mice were found to fire at stimuli (input current at 35 and 60pA, Fig 3D) lower than other groups. On the other hand, more than 80% of DRG neurons from all group were found to fire at injection current larger than 235 pA (Fig 3D, dashed line). Representative responses for DRG neurons from these WT and knock-in mice are shown in Fig 3E. The firing frequency, which is indicative of the average number of APs in 500 ms, was significantly higher in both in knock-in mice lines than in WT mice in response to a high input current stimulus (Fig 3F); firing frequency increased in F802C mice, the firing frequency increased with current injection, as previously reported for knock-in mice harboring the Nav1.9 p.R222S mutation [3], whereas it was constantly higher in F1125S mice than WT mice. These results suggest that DRG neurons of the F802C and F1125S mice show no differences in the shape of Aps and these mutations increased the firing frequencies when injected with constant currents. We conclude from these results that the DRG neurons of the F802C and F1125S knock-in mice have a higher level of excitability than DRG neurons of WT mice.

## Discussion

We previously identified *SCN11A* p.R222H/S mutations in six unrelated Japanese families with小児四肢疼痛発作症 [3]. In this study, we recruited additional potential patients with similar pain episodes over a two-year period from throughout Japan. We found that although these potential patients were distributed in various regions of Japan, the *SCN11A* p.R222H mutation was more frequent in the Tohoku area than in any other region. This suggests that genetic screening to detect p.R222H mutations for FEP syndrome is effective when the infant patients are suspected of having FEP, particularly in the Tohoku area. In addition to identifying two novel *SCN11A* mutations, p.F814C and p.F1146S, in this study, we also identified two previously-reported mutations, *SCN11A* p.R225C and p.V1184A [1, 2]. The clinical characteristics of the Japanese pedigrees carrying these p.R225C or p.V1184A mutations were almost the same as those of the previously-reported pedigrees [1, 2], although hyperhidrosis and gluten sensitivity could not be confirmed in the respective Japanese p.R225C and p.V1184A families. Thus, while these results suggest that ethnic differences have little effect on the pain symptoms, there is still some discordance in the autonomic symptoms. Furthermore, undiagnosed patient recruitment and genetic testing should be extended, as it is expected that there will be a considerable number of Japanese patients with小児四肢疼痛発作症 whose pain syndromes result from Nav1.9 mutations.

As Nav1.9 is preferentially expressed in small-diameter DRG neurons, which transmit pain to the spinal cord, mutations in Nav1.9 have generally been associated with painful or painless gain-of-function phenotypes [1–3,14–22]. The two newly-identified mutations of *SCN11A* mutations, p.F802C and p.F1125S, which were located at conserved regions among sodium channels (S1 Fig), significantly depolarized the RMP and increased the firing frequency in comparison with the WT, but had no significant effect on the current threshold or the AP parameters. It is of interest that the firing probability and frequency of F1125S was significantly increased at low stimuli compared with the WT, while firing frequency of F802C was significantly higher than WT at large stimuli without changing firing probability. It has been suggested that the Nav1.9 channel is not directly responsible for AP generation but rather that it is involved in modulation of nociceptor membrane potential as previously described [23–26]. Overall, we conclude that these novel Nav1.9 mutations, p.F814C and p.F1146S, cause FEP syndrome by raising the excitability of DRG neurons through mechanisms of gain-of-function mechanisms.

**Fig 4:**
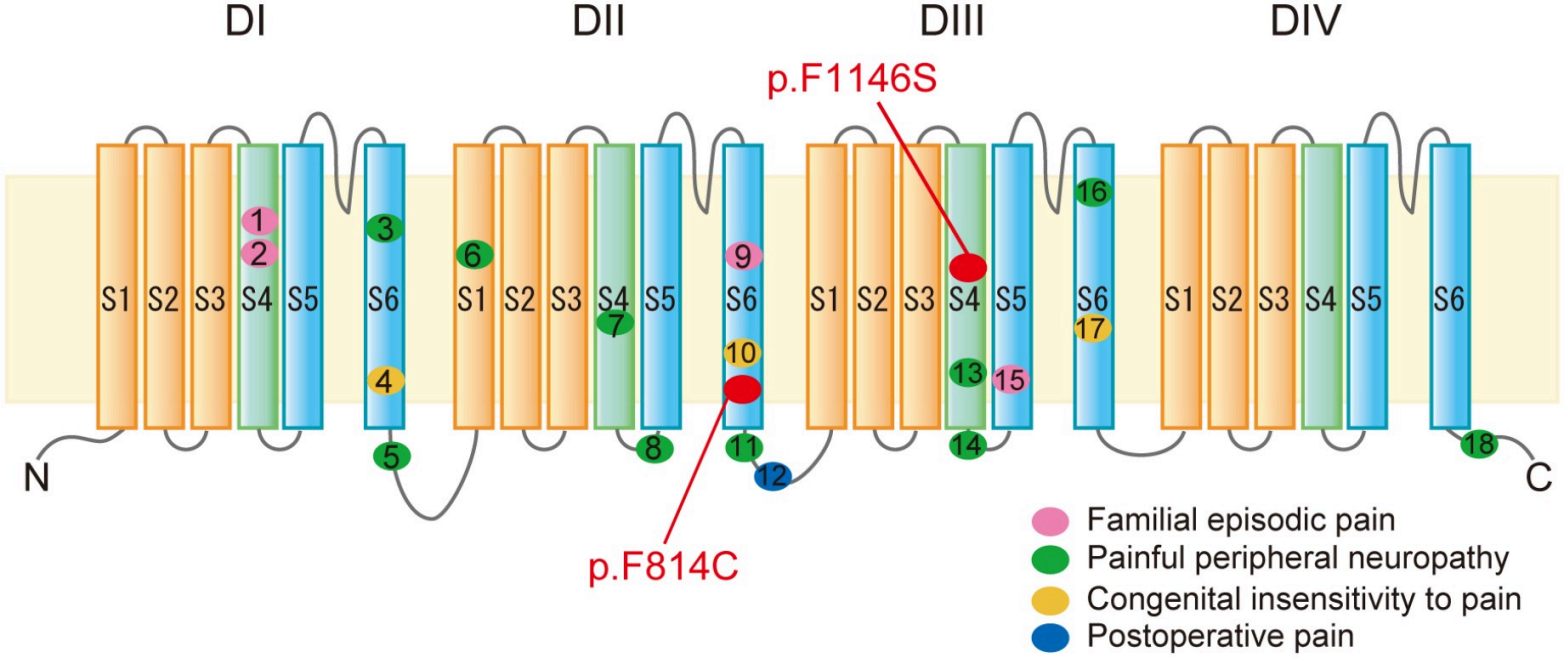
Schematic diagram of Nav1.9 and locations of mutations with pain disorders. Nav1.9 has four domains, each of which consistent of 6 transmembrane segments (S1-S6). S4 segment (S4: green segment) of each domain is voltage sensor, while the S5 and S6 segments (S5, S6: light blue segment) of each domain form the pore region of the voltage gated sodium channel. The novel Nav1.9 mutations p.F814C and p.F1146S are located in the S6 of DII and S4 in DIII. The numbered circles indicate the identified mutations causative of the pain disorders. Phenotypes of disorders are represented by color of the circles; pink, Familial episodic pain; green, Painful peripheral neuropathy; yellow, Congenital insensitivity to pain, blue; Postoperative pain. The numbered locations of each mutation (and their references) are follows: 1, R222H/S (3); 2, R225C (1); 3, I381T (19); 4, L396P (18); 5, K419N (19); 6, A582T (19); 7, A681D (19); 8, G699R (20); 9, A808G (1); 10, L811P (15); 11, A842P (19); 12, V909I (13); 13, L1158P (19); 14, N1169S (21); 15, V1184A (17); 16, I1293V (21); 17, L1302F (15); 18, F1689L (19).

The Nav1.9 mutations that have been reported to be related painful [1, 3, 17, 19–21] and painless [15, 18] disorders and sensitivity [13] were shown in Fig 4. While p. F814C is located at the lower of S6 transmembrane region in domain II, which is pore forming site. Nav1.9 p. F1146S is located at the middle of segment S4 transmembrane region in domain III, which is voltage sensor domain (VSD). Almost all the reported mutations related to painful and painless disorders are exclusively located S4-S6 segments, suggesting that these mutations may have influence on channel gating function. Recent elucidation of crystal structure of voltage-gated sodium channels [27–29] have revealed new insights into the mechanisms of gain-of function mechanisms [30], supporting a hypothesis that amino acid position at 814 and 1125, which are well preserved in sodium channels (S1 Fig), may alter the channel conformation during gating [27, 29]. Such theoretical conjectures on the gain-of-function mechanisms, however, are not been supported by experimental evidence: mutations located in the S6 segment reportedly have no influence on channel gating properties [1, 3]. Thus, further physiological studies, beyond just the gating properties, are needed to unequivocally determine the gain-of-function mechanisms. In fact, it was known Nav1.9 function is altered developmentally by glycosylation [31], modulated by other channels [32, 33] and activated by oxidized phospholipids under inflammatory conditions [34]. Therefore, further investigation are essential to explain the Nav1.9 gain-of-function mutations, and to lead to the development of novel therapeutic targets for FEP syndrome.

It has recently been demonstrated that mutations in Nav channels that result in neurogenic diseases impair the expression level or trafficking of the channels to the cell membrane [35–39]. Nav channels are composed of a pore-forming α subunit and their associated β subunits that modulate channel expression levels, voltage dependence and kinetics [40, 41]. In particular, the presence of β subunits is found to rescue channel activity by enhancing Nav channels expression and surface trafficking [42]. The contribution of β subunits to Nav1.9 expression and trafficking has not been addressed in this study, and so further experiments are needed to clarify this relationship. Furthermore, it is known that lowering the incubation temperature during Nav1.9 channel expression enhances surface trafficking [37, 40], and that disruption of Nav1.9 reduces pharmacologic induced pain and aggravated pain [43]. Patients in our study displayed in cold-induced sensitivity, suggesting the possibility that Nav1.9 may play a role in cold nociception, and that the Nav1.9 mutations found in this study may have modulate the cold-induced pain.

Our current study has two limitations. First, the functional expression of recombinant Nav1.9 in heterologous systems is historically difficult, and stable expression and characterization of recombinant Nav1.9 has proved challenging and, in many cases, unsuccessful. Such difficulties hamper the systematic investigation of channel properties [44]. Another limitation is that, although *SCN11A* gain-of-function mutations are reported to be associated with autonomic symptoms, such as hyperhidrosis and gastrointestinal dysfunction, we were unable to consistently confirm this association because of the indistinct segregation of autonomic symptoms in the pedigrees studied.

In conclusion, we have identified in the present study two novel mutations of *SCN11A* (p.F814C and p.F1146S) by recruitment of potential patients with similar FEP symptoms. Interestingly, our findings suggest that such Nav1.9 mutations contribute to a substantial proportion of FEP patients in Japan. Thus, future studies should also examine in more detail the proportion of FEP patients in Japan that resulting from such Nav1.9 mutations, and how these Nav1.9 mutations are distributed throughout Japan.

## Materials and methods

### Ethical statements

The clinical/genetic study on humans was approved by the Institutional Review Board and Ethics Committee of Kyoto University School of Medicine, Japan (approval no., G501; approval date, 2 August 2012), and Akita University Graduate School of Medicine, Japan (approval no., 960; approval date, 26 September 2012). Written informed consent was obtained from all subjects, and the parents of children and adolescents, before participation.

Animal studies, including animal care and all experimental procedures, were in accordance with the Animal Welfare Guidelines of Kyoto University. Animal experiment protocols were reviewed and approved by the Animal Care, Use and Ethics Committee at Kyoto University (approval nos., MedKyo16042 and MedKyo18523; and approval dates, 25 Mar 2016 and 3 May 2018, respectively).

### Patients and genomic DNA preparation

We raised a call to pediatricians at three meetings in Japan for suspected cases of 小児四肢疼痛発作症 with early-onset paroxysmal limb pain episodes. The meetings were as follows: The 120^th^ annual meeting of Japan Pediatric Society; the 58^th^ annual meeting of the Japanese Society for Inherited Metabolic Disease; and the 26^th^ annual meeting of the Pediatric Rheumatology Association of Japan. As a result, 42 unrelated Japanese families were recruited from March 2016 to March 2018. Peripheral blood was collected from 42 probands and 50 relatives (38 affected, 12 unaffected) from these families. Genomic DNA was extracted from whole blood samples using the QIAamp DNA Blood Mini Kit (Qiagen, Hilden, Germany).

### *SCN11A* mutation analysis

The scheme for *SCN11A* mutation screening in the present study is shown in Fig 1. We screened for *SCN11A* p.R222H and p.R222S mutations by Sanger sequencing in 41 pedigrees. When the p.R222H or p.R222S mutation was found in the proband and segregation was confirmed in the pedigree, we regarded it as causative *(SCN11A* p.R222H/S (+)). Subsequently, in probands with neither p.R222H nor p.R222S mutations (*SCN11A* p.R222H/S (-)), the entire *SCN11A* coding region and intron/exon boundaries were analyzed by Sanger sequencing. To distinguish deleterious variations from detected variations, we used the following series of filters: (1) non-synonymous variants (missense, nonsense, frameshift, and splice site variants); (2) known causative or novel mutations; (3) minor allele frequency (MAF) < 0.001 in the Japanese population from the 1000 Genomes database (phase 3) and a Japanese genetic variation database Human Genetic Variation Database (http://www.hgvd.genome.med.kyoto-u.ac.jp); and (4) variants present in affected members and not present in unaffected members of each pedigree (complete segregation).

Sanger sequencing for the entire *SCN11A* coding exons (including exon 6 in which p.R222H and p.R222S are located) was performed using primers described in our previous report [3] (S1 Table). Mutations were confirmed by both forward and reverse primers. Homology searches for sequence alignments with Nav family members were performed by BLAST (https://blast.ncbi.nlm.nih.gov/Blast.cgi).

### Exome analysis and Sanger sequencing

For Family 1, exome analysis was conducted. The exome analysis target region (exonic regions and flanking intronic regions) was captured using the SureSelect Human All Exon V5 Kit (Agilent Technologies, Santa Clara, CA, USA), and sequencing was performed using the Illumina HiSeq 1500/2500 platform (Illumina Inc., San Diego, CA, USA). Sequence reads were mapped to the reference human genome (UCSC Genome Browser hg19) using Burrows–Wheeler Aligner software.

The mutation identified by exome analysis was confirmed by Sanger sequencing.

### Nav1.9 knock-in mouse

Nav1.9 knock-in mice were generated as described previously [3]. In mice, the F1125S and F802C alterations are allelic orthologs of the human F1146S and F814C mutations, respectively. These mutations were introduced into mouse *Scn11a*, which correspond to each locus, using the CRISPR/Cas9 system by TransGenic Inc. (Fukuoka, Japan). Single guide RNAs (sgRNAs) targeting the regions around the mouse *Scn11a* of each locus was designed using the Optimized CRISPR Design web tool (http://crispr.mit.edu/) [45]. To avoid off-target effects, two sgRNAs were designed for each mutation. Both oligonucleotide DNAs encoding the sgRNAs (S2 Table) were synthesized, annealed, and cloned into the pX330-U6-Chimeric_BB-CBh-hSpCas9 plasmid [46] obtained from Addgene (Addgene plasmid #42230). Single-strand donor oligonucleotide DNA (donor oligoDNA), harboring the nucleotide variant that introduces the F1125S or F802C amino acid change, was synthesized (Integrated DNA Technologies, Coralville, IA, USA) (S2 Table). Each Cas9+sgRNA vector and donor oligoDNA were microinjected into fertilized C57BL/6 mouse eggs (originated from C57BL/6NCrSlc, CLEA Japan) to generate the two strains of *Scn11a* ^+/F1125S^ and *Scn11a* ^+/F802C^ mice. The nucleotide changes in genomic DNA corresponding to *Scn11a* F1125S and F802C were confirmed in offspring by direct sequencing using each of the primers described in S2 Table. Further genotyping was performed using TaqMan SNP genotyping assays (Applied Biosystems, Foster City, CA, USA).

### Isolation of DRG neurons

According to our previous report [3], DRG neurons were isolated from L4 to L6 sections of 6–8-week-old WT, F1125S and F802C mice. Briefly, these mice were euthanized by decapitation after being anesthetized with sevoflurane, and then transcardially perfused in artificial cerebrospinal fluid (aCSF (in mM); 124 NaCl, 5 KCl, 1.2 KH_2_PO_4_, 1.3 MgSO_4_, 2.4 CaCl_2_, 10 glucose, and 24 NaHCO_3_). DRG neurons were isolated with collagenase XI (Sigma-Aldrich, St.Louis, MO, USA) in incubation medium containing Earle’s balanced salt solution (Sigma-Aldrich) for 25 min at 37 °C. After collagenase digestion, isolated DRG neurons were resuspended in aCSF and plated onto non-coated 12 mm φ coverslips.

### Electrophysiology

Electrophysiological analysis was performed using a modification of a previously described protocol [3, 47]. Electrophysiological data from isolated DRG neurons were collected from small diameter (< 25 μm) cells from WT, F1125S and F802C mice. Data were obtained at 23 °C– 25 °C within 8 h after isolation using an EPC-9 amplifier (HEKA Elektronik, Lambrecht, Germany). Patch pipettes were fabricated from thin-walled borosilicate glass capillaries (GC150TF-10, Harvard Apparatus, MA, USA), and had a resistance of 1.5–2.5 MΩ. Patch pipette tips were fire-polished before use. Electrode capacitance was compensated electrically, and series resistance was < 13 MΩ and compensated by 55–80%. Data were collected in cells that required a smaller current than -30 pA to hold the membrane at -60 mV. Cell viability was monitored by RMP and input resistance, the cell which RMP varied more than 10% and input resistance varied more than 10 MΩ were exclude from data analysis. If the series resistance changed by more than 25%, the cell was also excluded from analysis. According to our previous study [3], current-clamp recordings were obtained after achieving more than 5 min of whole-cell recording conditions. The pipette solution contained (in mM): 67 KCl, 65 K-gluconate, 1 MgCl_2_, 5 EGTA, 4 ATP-Mg, 1 GTP-Na_2_, and 10 HEPES (pH 7.3 with KOH). The bath solution for isolated DRG neurons contained (in mM): 130 NaCl, 5 KCl, 1 MgCl_2_, 2 CaCl_2_, 10 glucose, and 10 HEPES (pH 7.4 with NaOH). RMP was measured at *I* = 0 before current injection in every trial. The following parameters of the first AP were measured: amplitude, 50% AP width, and maximum rate of rise/fall of AP. To evaluate the input impedance, the voltage response amplitude was measured at a current injection of 10 pA. Current threshold was defined by the lowest input current which induced an action potential in DRG neurons. We observed firing of each DRG neuron in response to the step current injections (500ms) from 10pA in 25 pA increments and determined current threshold for each cell. We then calculated means and SDs for three groups. For firing probability at a given input current, we observed firing DRG neurons in response to a current stimulus (500ms) from 10 to 285 pA in 25 pA increments and calculated the firing probability by dividing the firing cell numbers by total of firing and non-firing cells at a given input current. Firing frequency was calculated from the AP number during step current injections (500 ms) from 10 to 235 pA in 25 pA increments. Cells that did not generate APs, or had only one AP in response to a 500-ms current stimulus in all step pulses, were excluded from the analysis. All data were acquired using Patchmaster (HEKA Elektronik). Electrophysiological data of isolated DRG neurons were analyzed using Igor Pro (WaveMetrics Inc., Portland, OR, USA).

### Statistical analysis

Electrophysiological data are presented as mean ± standard error of the mean (S.E.M.). Statistical analyses for RMP, the input impedance, the various AP parameters, current threshold were first tested by one-way ANOVA. When one-way ANOVA was significant (i.e., p<0.05), we then conducted the Student’s *t*-test with Bonferroni correction between WT and F802C mice or between WT and F1125S mice. For statistical significance, the p value was corrected by the Bonferroni method (i.e., 0.05/2) to maintain the statistical significance level at 0.05 in multiple comparisons (comparisons between WT and F802C mice, and between WT and F1125S mice). The firing probability were compared among the three groups by Fisher’s exact test and, when it was significant, then between WT and F802C or between WT and F1125S by Fisher’s exact test with the Bonferroni method. The firing frequency was compared among the three groups by Kruskal-Wallis test and, when it was significant, then by Dunn’s multiple comparisons test. All statistical analyses were performed using SAS software (Version 9.4; SAS Institute, Cary, NC, USA). Statistical significance is indicated by *, p<0.05, and**, p<0.01.

## Supporting information

S1 Fig Conservation of p.F814C and p.F1146S mutations compared with Nav1.1—Nav1.8. The accession numbers of the Nav channels shown are as follows: Nav1.1, NP_001159435.1; Nav1.2, NP_066287.2; Nav 1.3, NP_008853.3; Nav1.4, NP_000325.4; Nav1.5, NP_001092874.1; Nav1.6, NP_055006.1; Nav1.7, NP_002968.1; Nav1.8, NP_006505.3; Nav1.9, NP_001336182.1.
(PDF)

S1 Table Primers used for amplification of *SCN11A*. * denotes the primer used for sequencing of each mutation.
(XLS)

S2 Table Oligonucleotides and primers used for production of Nav1.9 knock-in mice lines. (XLS)
