# Supplementary material for: Familial episodic limb pain in kindreds with novel Nav1.9 mutations
Source: PLoS One. 2018 Dec 17;13(12):e0208516. doi: 10.1371/journal.pone.0208516 (PMC6296736; doi:10.1371/journal.pone.0208516)
Supplement: S1 Fig — The accession numbers of the Nav channels shown are as follows: Nav1.1, NP_001159435.1; Nav1.2, NP_066287.2; Nav 1.3, NP_008853.3; Nav1.4, NP_000325.4; Nav1.5, NP_001092874.1; Nav1.6, NP_055006.1; Nav1.7, NP_002968.1; Nav1.8, NP_006505.3; Nav1.9, NP_001336182.1. (PDF) [file pone.0208516.s001.pdf]

	p.F814C	p.F1146S
Nav1.9 (SCN11A)	LLLNSFSNEER	MELKSFRTLRA
Nav1.1 (SCN1A)	LLLSSFSADNL	GAIKSLRTLRA
Nav1.2 (SCN2A)	LLLSSFSSDNL	GAIKSLRTLRA
Nav1.3 (SCN3A)	LLLSSFSSDNL	GAIKSLRTLRA
Nav1.4 (SCN4A)	LLLSSFSADSL	GPIKSLRTLRA
Nav1.5 (SCN5A)	LLLSSFSADNL	GPIKSLRTLRA
Nav1.6 (SCN8A)	LLLSSFSADNL	GAIKSLRTLRA
Nav1.7 (SCN9A)	LLLSSFSSDNL	GPIKSLRTLRA
Nav1.8 (SCN10A)	LLLNSFSADNL	APIKALRTLRA
